# Metastatic follicular thyroid cancer with a longstanding responsiveness to gemcitabine plus oxaliplatin

**DOI:** 10.1530/ETJ-22-0227

**Published:** 2023-05-08

**Authors:** Daniela Dias, Inês Damásio, Pedro Marques, Helder Simões, Ricardo Rodrigues, Branca Maria Cavaco, Valeriano Leite

**Affiliations:** 1 Endocrinology Department, Instituto Português de Oncologia de Lisboa Francisco Gentil, Lisbon, Portugal; 2 Endocrinology Department, Hospital de Santa Maria, Centro Hospitalar Universitário de Lisboa Norte (CHULN), Lisbon, Portugal; 3 Unidade de Investigação em Patobiologia Molecular (UIPM), Instituto Português de Oncologia de Lisboa Francisco Gentil, Lisbon, Portugal; 4 Nova Medical School: Faculdade de Ciências Médicas da Universidade Nova de Lisboa, Lisbon, Portugal

**Keywords:** advanced follicular thyroid carcinoma, GEMOX (gemcitabine plus oxaliplatin), multikinase inhibitors

## Abstract

**Background:**

Treatment of advanced follicular thyroid carcinoma (FTC) is based primarily on indirect evidence obtained with multikinase inhibitors (MKI) in clinical trials in which papillary carcinomas represent the vast majority of cases. However, it should be noted that MKI have a non-negligible toxicity that may decrease the patient’s quality of life. Conventional chemotherapy with GEMOX (gemcitabine plus oxaliplatin) is an off-label therapy, which seems to have some effectiveness in advanced differentiated thyroid carcinomas, with a good safety profile, although further studies are needed.

**Case report:**

We report a case of a metastatic FTC, resistant to several lines of therapy. However, with a durable response to GEMOX, the overall survival of our patient appears to have been extended significantly due to this chemotherapy.

**Conclusion:**

GEMOX may have a role in patients with thyroid cancer unresponsive to MKI.

## Established facts

- Patients with radioactive iodine-refractory differentiated thyroid cancer account for two-thirds of patients with distant metastasis.
- In the past years, novel targeted therapies directed to specific genes have been developed for advanced thyroid carcinoma.
- Multikinase inhibitors have a non-negligible toxicity that may decrease the patient’s quality of life.

## Novel insights

- GEMOX (gemcitabine plus oxaliplatin) may have a role in patients with thyroid cancer unresponsive to multikinase inhibitors.

## Introduction

The majority of thyroid cancers of follicular origin have an excellent prognosis. However, less than 10% of cases will have an aggressive behavior, developing distant metastatic or advanced loco-regional disease. Most of these aggressive cases are radioiodine refractory (RAIR), making RAI treatment (RAIT) obsolete in such cases. When the disease is RAIR, the average patient survival is less than 5 years. Significant progress in the treatment of advanced thyroid carcinomas has occurred in the past years with the development of multikinase inhibitors (MKIs) (1, 2, 3). The Food and Drug Administration (FDA) and the European Medicines Agency (EMA) have approved the MKIs sorafenib and lenvatinib for the treatment of metastatic progressive differentiated thyroid cancer (DTC) of follicular origin. These drugs now play a major role as first-line targeted therapy for such tumors. Progression-free survival was 10.8 months in patients with advanced DTC receiving sorafenib (vs 5.8 months in the placebo group) and 18.3 months with lenvatinib (vs 3.6 months in the placebo group) (4, 5). In 2021, the FDA approved cabozantinib for locally advanced and metastatic DTC that has progressed following VEGFR-targeted therapy (6). Sunitinib is still used as an *off-label* therapy in these patients based on results from phase II trials (7, 8, 9). Recently, drugs targeting specifically genes such as *BRAF, RET,* and *NTRK* have also shown high efficacy in advanced thyroid tumors, with less toxicity than MKIs (3, 10, 11, 12). Conventional cytotoxic chemotherapy has shown poor efficacy and high toxicity and has been replaced with more efficient therapeutic modalities such as tyrosine kinase inhibitors and molecular targeted therapies (4, 5, 6, 10, 11, 12, 13, 14). Despite these new agents, cytotoxic chemotherapy still has a role in selected patients with RAIR tumors (12, 15). *Spano et al.* conducted a retrospective analysis of gemcitabine plus oxaliplatin (GEMOX) in RAIR thyroid cancer (16). Six of 14 patients had a diagnosis of follicular thyroid carcinoma (FTC). An overall response rate of 57% and a progression-free survival of 10 months was observed with an objective response rate in all FTCs. A phase II trial was initiated to evaluate the efficacy and tolerance of GEMOX in RAIR thyroid cancer; however, it was terminated early due to inefficiency (https://clinicaltrials.gov/ct2/show/NCT02472080). This therapeutic regimen has shown beneficial effects in advanced cancers of different tissues such as pancreas, liver, ovary and lung, and in Hodgkin’s lymphoma (16, 17, 18, 19, 20). We present a case of a metastatic FTC unresponsive to three lines of MKI and to chemotherapy with doxorubicin/docetaxel, but with a durable response to GEMOX.

## Case report

**Figure 1:**
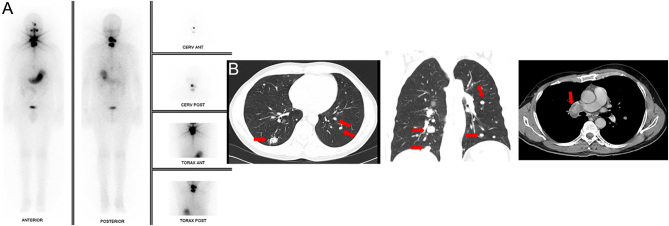
(A) Whole-body scan after ^131^I treatment revealed moderate cervical uptake. (B) Lung, mediastinal, and hilar lymph nodes metastasis after total thyroidectomy and radioiodine treatment.

**Figure 2:**
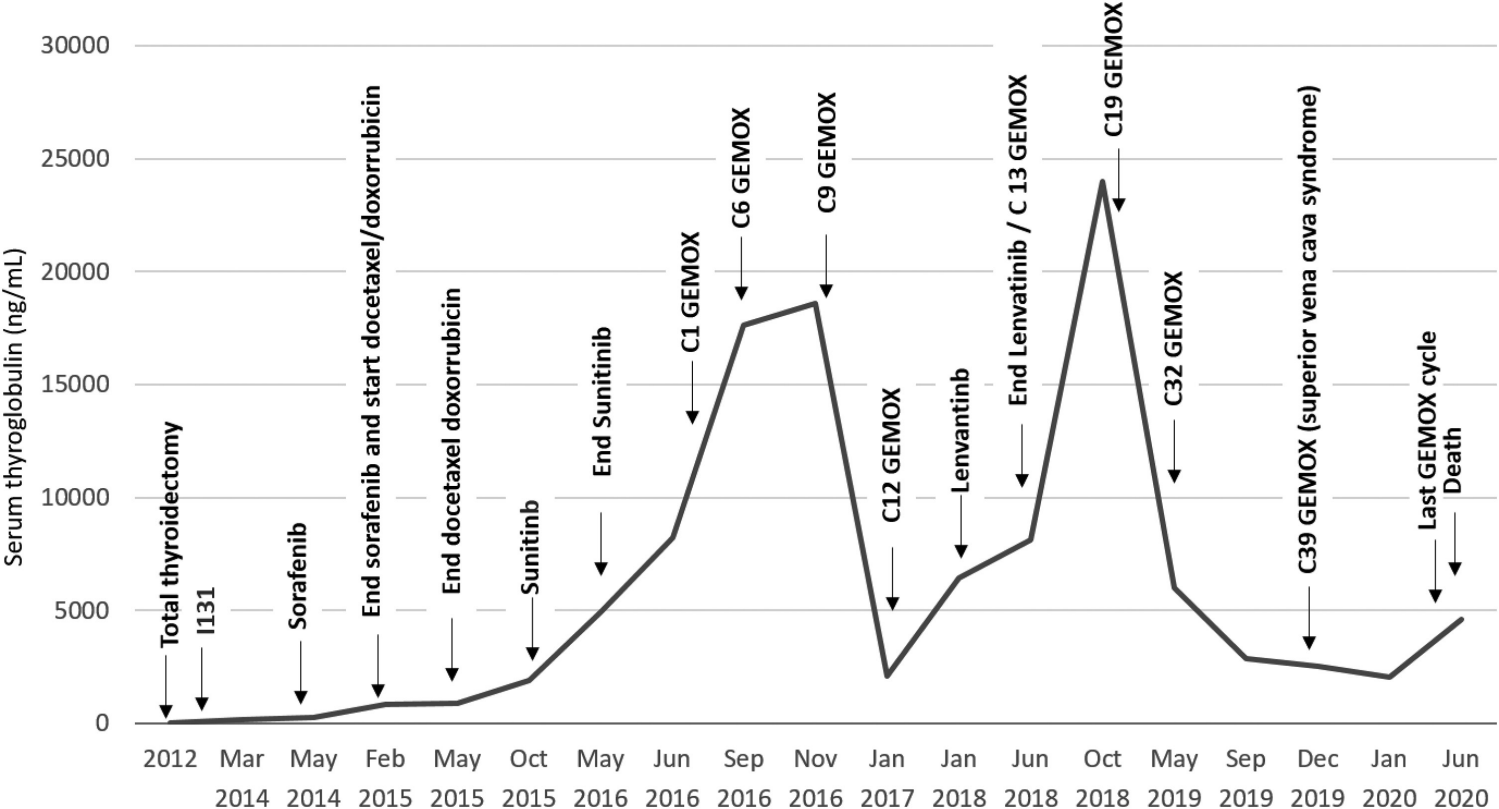
Pattern of serum Tg evolution during the course of the disease. Gemox induced a transient increase in serum thyroglobulin (sTg) levels, followed by a sustained decrease. C= cycle.

**Figure 3:**
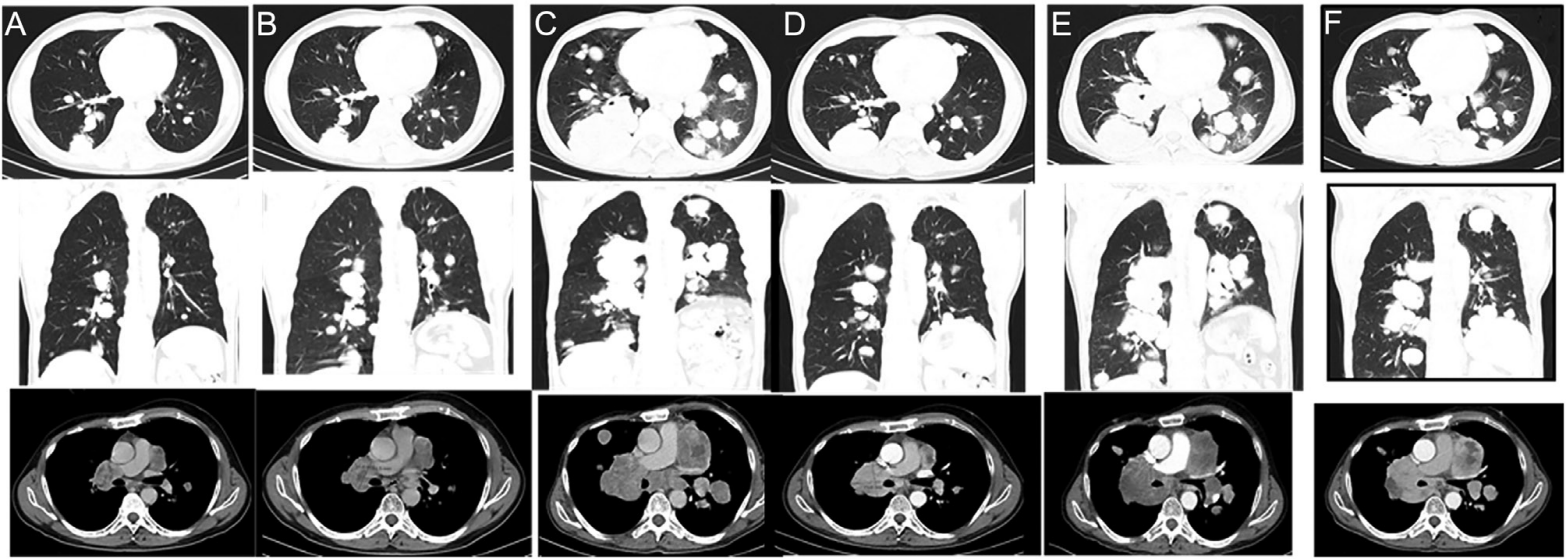
CT findings in the chest during treatment. Evaluation after sorafenib (A), docetaxel/doxorubicin (B), sunitinib (C), 12 cycles with GEMOX (D), lenvantinib (E), and 19 additional cycles of GEMOX (F).

A 52-year-old man was referred to our institution in 2012, two years after a total thyroidectomy for an FTC. The histopathological analysis showed a 16 mm FTC, with vascular invasion and 50% of poorly differentiated areas (pT1b pNx Mx). Post-operative staging revealed a basal serum thyroglobulin (sTg) of 9 ng/mL, negative anti-Tg antibodies, and a micronodule in the left lung in a CT scan. He was asymptomatic and had a good performance status. The patient received 100 mCi of RAI in 2013, and a post-treatment whole-body scan revealed moderate cervical uptake (Fig. 1A), with no distant uptake foci. Stimulated sTg was 408 ng/mL. Sixteen months later (2014), suppressed sTg raised up to 183 ng/mL (Fig. 2), and disease progression in the lungs, as well as in the mediastinal and hilar lymph nodes, was observed (Fig. 1B). His disease was considered RAIR, and he was then commenced on sorafenib in May 2014, with 400 mg twice daily. He developed high blood pressure, which was well-controlled with losartan/hydrochlorothiazide, grade 2 asthenia, anorexia, and diarrhea. The dosage of sorafenib was reduced to 600 mg daily. Sorafenib was discontinued in February 2015 due to biochemical (sTg level raised to 827 ng/mL; Fig. 2) and structural progression of the disease (Fig. 3A). He was treated with second-line chemotherapy, with five cycles of docetaxel (60 mg/m^2^) and doxorubicin (50 mg/m^2^) from February to May 2015. Serum Tg at the end of treatment was 893 ng/mL (Fig. 2), and a CT scan showed progression of metastatic disease in the mediastinal lymph nodes and lungs (Fig. 3B). In September 2015, significant disease progression in these tissues was observed and a new lytic lesion in the right iliac was demonstrated by FDG-PET/CT, accompanied by a significant rise in sTg up to 1881 ng/mL (Fig. 2). In October 2015, the bone lesion was treated with 30 Gy of palliative external-beam radiation, and sunitinib 50 mg daily (4 weeks on, 2 weeks off) was started. However, just after one cycle, sunitinib was suspended for 2 months due to poor tolerability (grade 2 anorexia, fatigue, and hemoptoic sputum). Bronchoscopy showed a vascular endobronchial lesion in the right upper bronchus and in the apical bronchus of the right lower lobe. He resumed sunitinib at a lower dose (37.5 mg) once daily, continuously. Six months later, worsening of dyspnea, unresponsive to bronchodilators and corticosteroids, with a permanent requirement of oxygen supplementation, was observed. Serum Tg level increased to 4926 ng/mL (Fig. 2), and marked structural progression was seen on chest CT scan (Fig. 3C). The patient was then considered for *off-label* therapy with gemcitabine 1000 mg/m^2^ plus oxaliplatin 100 mg/m^2^ (GEMOX), every two weeks (16). GEMOX was started in July 2016. After nine cycles, due to grade 3 feet sensory neuropathy, the interval between cycles was prolonged (every 3 weeks) and oxaliplatin dosage was reduced by 20%. He completed three additional cycles (the last in January 2017). A favorable clinical (improvement of dyspnea allowing discontinuation of oxygen supply), biochemical (sTg decreased from 8207 ng/mL to 2086 ng/mL despite an initial increase up to a maximum of 18605 ng/mL, Fig. 2), and structural response (Fig. 3D) was observed. The best objective response was a 46% partial response, which was obtained 6 months after starting GEMOX and was sustained for 7 months after GEMOX discontinuation in January 2017. He remained asymptomatic for approximately 12 months. In February 2018, due to worsening of respiratory symptoms, lenvatinib, at a daily dosage of 24 mg, was initiated but discontinued after 4 months because of disease progression (Fig. 3E). Once again, disease stability was only achieved with GEMOX, which was reinstituted in June 2018 for additional 27 cycles (Fig. 3F), with each cycle being administered every 2–3 weeks (depending on blood counts). However, in December 2019, he presented with superior vena cava syndrome and stent implantation, and radiotherapy and enoxaparin were required. In January 2020, mutational profiling of the tumor was performed through Sanger sequencing for *TERT* promoter (*TERT*p), and next-generation sequencing (NGS) for 52 solid tumors’ related genes. Briefly, NGS was performed with DNA and RNA from the patient’s FTC, using the AmpliSeq^TM^ for Illumina Focus Panel (Illumina, CA, USA), which allows the analysis of single nucleotide variants (SNVs), indels, copy number variations (CNVs), and gene fusions. The Variant Interpreter software (Illumina) was used for variant annotation. One variant in the *TP53* gene (transcript NM_000546.5, c.659A>C p.Tyr220Ser) was identified, showing a high variant allele frequency (VAF = 68.8%), suggesting possible hemizygosity due to loss of the wild-type allele. The Tyr amino acid residue is conserved in several species, including *Musmusculus* and *Xenopustropicalis*, and *in silico* characterization with Sift and PolyPhen showed that this change was predicted to be deleterious and probably damaging, respectively. Its clinical significance was described to be likely pathogenic in ClinVar (VCV000012383.7) and pathogenic in COSMIC (COSM43850). No additional genetic alterations were identified. Gene fusions could not be assessed due to poor RNA quality.

Because no druggable target was identified, six additional GEMOX cycles, every 3 weeks, were administered that resulted in improvement of respiratory symptoms. But, in June 2020, he was hospitalized for pneumonia. During this hospital stay, he had a traumatic brain injury after falling from his own height and died 2 days later. It is worth to mention that, in this second period of GEMOX treatment (June 2018 to June 2020), the best response was stable disease (the sum of diameters of the target lesions decreased by 24%). At the last imaging evaluation (CT scan), just 2 months before the patient’s death, in June 2020, and compared to the CT scan at the initiation of GEMOX treatment in July 2016, there was a 37% structural response.

## Discussion

Follicular thyroid carcinoma (FTC) is a rare cancer, corresponding to less than 10% of all differentiated thyroid cancer (DTC). Compared to classic papillary thyroid carcinoma (PTC), FTC displays a different clinical behavior and genetic profile (21, 22, 23). A hematogenous metastatic pattern is generally observed, more commonly involving the lungs and bones (1, 2, 23).

*RAS* mutations are the most prevalent in FTC (30–50%), while one-third of cases may have *PAX8/PPARγ* rearrangements (23, 24). In the present case, no *RAS* mutations were detected, and gene fusions could not be assessed. *TERT*p mutations, which have been described in approximately 15% of FTCs and associated with worst clinical and prognostic features (25, 26), were absent in the tumor. However, a mutation in the* TP53* tumor suppressor gene was identified, which has been reported in 28.6% of FTC and in 0% of follicular thyroid adenomas (FTA) (27).

FTC usually represents a minority of tumor types included in trials with MKIs. For instance, in the sorafenib arm of the Decision trial only 13 cases (6.3%) were non-Hürthle FTC, while in the Select trial there were only 53 patients (20.3%) with non-Hürthle FTC in the lenvatinib arm (4, 5). Hence, therapeutic strategies for FTC are based primarily on indirect evidence obtained with PTC. *Spano et al.* (16) reported promising results with the GEMOX regimen every 2 weeks for 12 cycles in RAIR thyroid cancer. In this study, the overall response rate was 57% and there was no major toxicity. The most common treatment-related adverse events were asthenia, peripheral neuropathy, diarrhea, anemia, thrombocytopenia, and neutropenia. In our patient, GEMOX was highly effective in inducing an objective response, which was maintained for 4 years with acceptable tolerability. It is worth mentioning that we administered a much higher number of cycles (*n* = 45) to our patient than the 12 cycles proposed by *Spano et al.* (16). Hematological toxicity and peripheral neuropathy were seen in our patient; however, the benefits in his overall survival and quality of life outweighed the side effects.

In a study performed by *Genutis et al.* (28), microsatellite instability (MSI) was reported in 2.5% of FTC cases. When present, MSI can be an indication for immune checkpoint inhibitor treatment (29). However, published data shows that immunotherapy has a modest antitumor activity in differentiated thyroid cancer (30). In addition, microsatellite instability was not identified in the tumor, so it is unlikely that immunotherapy could represent an option for this patient. Whether tumors with* TP53* mutations, such as in our patient, have a better response to GEMOX than *TP53*-negative tumors remains to be determined. In addition, other molecular alterations, not addressed in the present study, may also account for the good response observed.

In conclusion, cytotoxic chemotherapy with GEMOX may constitute a good option in patients with RAIR advanced thyroid cancer that is unresponsive to MKIs. Further studies are needed to understand the specific role of GEMOX chemotherapy in RAIR thyroid carcinomas and the impact of the associated molecular profiles.

## Declaration of interest

The authors declare that there is no conflict of interest that could be perceived as prejudicing the impartiality of this case report.

## Funding

Ricardo Rodrigues is a recipient of a PhD scholarship by iNOVA4Health Research Unit (UIDP/04462/2020; UI/BD/154256/2022), a program co-funded by Fundação para a Ciência e a Tecnologia, Portugal, and by Ministério da Ciência e do Ensino Superior, Portugal. This work was funded by Associação de Endocrinologia Oncológica (AEO) and by Instituto Português de Oncologia de Lisboa Francisco Gentil (IPOLFG).

## Statement of ethics

The patient’s daughter provided written informed consent for this case report.

## Author contribution statement

All authors made substantial contributions to this study, and all authors approved the final version of the manuscript.
